# German guidelines on community-acquired acute bacterial meningitis in adults

**DOI:** 10.1186/s42466-023-00264-6

**Published:** 2023-08-31

**Authors:** Matthias Klein, Carsten Abdel-Hadi, Robert Bühler, Beatrice Grabein, Jennifer Linn, Roland Nau, Bernd Salzberger, Dirk Schlüter, Konrad Schwager, Hayrettin Tumani, Jörg Weber, Hans-Walter Pfister

**Affiliations:** 1 Department of Neurology, LMU Klinikum, Ludwig-Maximilians-University, Munich, Germany; 2 Emergency Department, LMU Klinikum, Ludwig-Maximilians-University, Munich, Germany; 3 Munich, Germany; 4 Department of Neurology, Bürgerspital, Solothurn, Switzerland; 5 Klinische Mikrobiologie und Krankenhaushygiene, LMU Klinikum, Ludwig-Maximilians-University, Munich, Germany; 6 Department of Diagnostic and Interventional Neuroradiology, Faculty of Medicine and University Hospital Carl Gustav Carus, Dresden University of Technology, Dresden, Germany; 7 Department of Neuropathology, Evangelisches Krankenhaus Göttingen-Weende, Georg-August-University, Göttingen, Göttingen, Germany; 8 Klinik und Poliklinik für Innere Medizin, University of Regensburg, Regensburg, Germany; 9 Institut für Medizinische Mikrobiologie und Krankenhaushygiene, Medizinische Hochschule, Hannover, Germany; 10 Klinik für Hals-Nasen-Ohrenkrankheiten, Kopf- Hals- und plastische Gesichtschirurgie, Kommunikationsstörungen, Fulda, Germany; 11 Labor für Liquordiagnostik, Neurologische Universitätsklinik Ulm, University of Ulm, Ulm, Germany; 12 Department of Neurology, Klinikum Klagenfurt, Klagenfurt, Austria

**Keywords:** Acute bacterial meningitis, Streptococcus pneumoniae, Neisseria meningitidis, Listeria monocytogenes, Meningitis, Central nervous system infection, Guideline

## Abstract

**Introduction:**

The incidence of community-acquired acute bacterial meningitis has decreased during the last decades. However, outcome remains poor with a significant proportion of patients not surviving and up to 50% of survivors suffering from long-term sequelae. These guidelines were developed by the *Deutsche Gesellschaft für Neurologie* (DGN) under guidance of the *Arbeitsgemeinschaft der Wissenschaftlichen Medizinischen Fachgesellschaften* (AWMF) to guide physicians through diagnostics and treatment of adult patients with acute bacterial meningitis.

**Recommendations:**

The most important recommendations are: (i) In patients with suspected acute bacterial meningitis, we recommend that lumbar cerebrospinal fluid (with simultaneous collection of serum to determine the cerebrospinal fluid-serum glucose index and blood cultures) is obtained immediately after the clinical examination (in the absence of severely impaired consciousness, focal neurological deficits, and/or new epileptic seizures). (ii) Next, we recommend application of dexamethasone and empiric antibiotics intravenously. (iii) The recommended initial empiric antibiotic regimen consists of ampicillin and a group 3a cephalosporin (e.g., ceftriaxone). (iv) In patients with severely impaired consciousness, new onset focal neurological deficits (e.g. hemiparesis) and/or patients with newly occurring epileptic seizures, we recommend that dexamethasone and antibiotics are started immediately after the collection of blood; we further recommend that —if the imaging findings do not indicate otherwise —a lumbar CSF sample is taken directly after imaging. (v) Due to the frequent occurrence of intracranial and systemic complications, we suggest that patients with acute bacterial meningitis are treated at an intensive care unit in the initial phase of the disease. In the case of impaired consciousness, we suggest that this is done at an intensive care unit with experience in the treatment of patients with severe CNS diseases.

**Conclusions:**

The German S2k-guidelines give up to date recommendations for workup, diagnostics and treatment in adult patients with acute bacterial meningitis.

## Introduction

The main causative pathogens in adults with acute bacterial community-acquired meningitis are *Streptococcus pneumoniae, Neisseria meningitidis*, and *Listeria monocytogenes*. As a result of the available vaccinations against *Haemophilus influenzae* and *Neisseria meningitidis*, the incidence of acute bacterial meningitis has been declining over the last 30 years. In contrast, pneumococcal vaccinations have not resulted in a strong reduction in the incidence of pneumococcal meningitis in adults. Interestingly, during the first 2 years of the corona pandemic, there was a significant decrease in meningitis cases caused by *Streptococcus pneumoniae* in Germany. The number of invasive meningococcal diseases was also significantly reduced compared to previous years.

Acute bacterial meningitis remains a severe disease with unfavorable outcome in a large proportion of patients. Early recognition of patients with suspected acute bacterial meningitis, quick workup and early initiation of antibiotics have shown to be key factors that influence outcome. The German S2k- guideline gives up to date recommendations for workup, diagnostics and treatment in adult patients with acute bacterial meningitis.

This paper is a an abridged and translated short version of the German S2k-guideline on acute bacterial meningitis *AWMF number 030/089*.

## The most important recommendations at a glimpse

- In patients with suspected acute bacterial meningitis, we recommend that lumbar cerebrospinal fluid (with simultaneous collection of serum to determine the cerebrospinal fluid-serum glucose index and blood cultures) is obtained immediately after the clinical examination (in the absence of severely impaired consciousness, focal neurological deficits, and/or new epileptic seizures).
- Next, we recommend application of dexamethasone and empiric antibiotics intravenously.
- The recommended initial empiric antibiotic regimen consists of ampicillin and a group 3a cephalosporin (e.g., ceftriaxone).
- In patients with severely impaired consciousness, new onset focal neurological deficits (e.g. hemiparesis) and/or patients with newly occurring epileptic seizures, we recommend that dexamethasone and antibiotics are started immediately after the collection of blood; we further recommend that —if the imaging findings do not indicate otherwise —a lumbar CSF sample is taken directly after imaging.
- Due to the frequent occurrence of intracranial and systemic complications, we suggest that patients with acute bacterial meningitis are treated at an intensive care unit in the initial phase of the disease. In the case of impaired consciousness, we suggest that this is done at an intensive care unit with experience in the treatment of patients with severe CNS diseases.

## Guidelines in detail

### Definition, clinic presentation, and epidemiology

The main clinical symptoms of bacterial (purulent) meningoencephalitis are headache (83–87%), meningism (75–83%), impaired consciousness (69%), and fever (77%). Typical clinical symptoms of meningitis may be absent or only mild - a combination of three of the four cardinal symptoms is present in only half of the patients [1, 2]. Furthermore, nausea, vomiting, photophobia, confusion, and epileptic seizures can initially occur [2–4].

The incidence of acute bacterial meningitis has been declining in Europe over the past 30 years. A recent publication from the Netherlands showed that the incidence fell from 6.37 to 100,000 population (1989 to 1993) to 1.58 per 100,000 population per year (2014 to 2019). The background discussed above all is a decline in meningitis caused by *Haemophilus influenzae* type B and *Neisseria meningitidis* as a result of corresponding vaccination campaigns [5]. On the other hand, the introduction of pneumococcal vaccinations seems to have led to a decline in vaccine-preventable pneumococcal serotypes [6]; at the same time, however, there was an increase in cases of pneumococcal meningitis due to serotypes not covered by the vaccines (serotype replacement) [7].

The most common pathogens causing bacterial meningoencephalitis in adults in Central Europe are *Streptococcus pneumoniae*, followed by *Neisseria meningitidis* and *Listeria monocytogenes* (< 10% of cases), staphylococci (1–9% of cases depending on the literature), Gram-negative Enterobacterales, and *Pseudomonas aeruginosa* (< 10% of cases) and *Haemophilus influenzae* (1–3%) [7, 8]. The frequency of an infection due to *Listeria monocytogenes* is significantly higher in elderly patients, patients with malignancies, and patients receiving immunosuppressive therapy [8, 9]. The most common causative agents of purulent meningoencephalitis in childhood are pneumococci and meningococci, in the neonatal period *Streptococcus agalactiae* (group B streptococci), *Escherichia coli* and *Listeria monocytogenes*.

#### Recommendation 1 (strong consensus)

The cardinal symptoms of bacterial meningitis are: headache, meningism, fever, and impaired consciousness. We recommend that acute bacterial meningitis is considered if a combination of the before-mentioned symptoms is present. The absence of individual cardinal symptoms does not rule out bacterial meningitis.

#### Recommendation 2 (strong consensus)

In community-acquired acute bacterial meningitis in adults in Europe, the pathogens *Streptococcus pneumoniae* followed by *Neisseria meningitidis* and *Listeria monocytogenes* should be considered.

### Diagnosis

In untreated, immunocompetent patients with acute bacterial meningitis, the CSF is usually purulent-cloudy. It typically shows granulocytic pleocytosis with more than 1000 cells/µl, severe blood-CSF barrier dysfunction (total protein > 1000 mg/dL) and a decrease in CSF glucose (usually < 30 mg/dL; CSF/serum glucose quotient < 0.3) or an increase in CSF lactate (> 3.5 mmol/L) (Table 1) [10–12]. Depending on the pathogen, however, “atypical” findings are found in up to one third of cases: In a study of 1816 patients with pneumococcal meningitis, the cell count was < 1000/µl in 33% of patients [13].

**Table 1:** CSF Parameters in Meningitis^1^

Parameter in CSF	bacterial [13]	viral [38]	tuberculous [39]
**cell count/µl**	> 1000	< 1000	< 1000
**cytology**	granulocytes	lymphocytes ^2^	mixed
**CSF-serum-glucose-index**	decreased	normal	moderately decreased
**lactate (mmol/l)**	> 3,5	< 3,5	> 3,5
**total protein (mg/dL)**	> 100	< 100	> 100
**blood-CSF-barrier (CSF-serum albumin ratio)**	severely disrupted	normal	severely disrupted
**intrathecal Ig-synthesis**	IgA, IgG	IgG	IgA

^1^The actual CSF constellations can differ, especiall in early stages of the disease. E.g., one third of the patients with acute bacterial meningitis present with a CSF cell count of < 1000/µl [13]. ^2^Granulocytes can dominate in early stages of a viral infections (especially in adults) [38]

In clinical trials, testing serum procalcitonin has been helpful in distinguishing between bacterial and viral meningitis in children and adults [14–17]: A sensitivity of almost 99% with a specificity of over 80% or a sensitivity of 95% with a specificity of 100% was found. However, the cutoffs recommended depended on which assay was used and whether a high sensitivity or a high specificity was prioritized. Especially in the early phase of the disease, procalcitonin is more sensitive than C-reactive protein.

If there is a persistent suspicion of bacterial meningitis after CSF laboratory tests have been carried out, immediate confirmation of the diagnosis should be sought by detecting the pathogen in the CSF and in the blood (blood culture). With respect to CSF the following methods are suitable for this:

- microscopically using Gram stain (or methylene blue stain),
- molecular genetics using PCR (single-PCR, multiplex-PCR),
- bacteriologically by culture, and.
- latex agglutination tests (in special situations, explanation see below).

Current PCR test systems now allow rapid identification of pathogens within less than 2 h. Multiplex PCR meningitis panels, which detect the most common viral and bacterial pathogens of meningitis and encephalitis, show high sensitivities with high specificities, especially for the detection of bacterial pathogens (in a meta-analysis for *Streptococcus pneumoniae*, *Neisseria meningitidis*, *Haemophilus influenzae*, *Escherichia coli* and *Listeria monocytogenes* sensitivity was 89.5% and specificity 97.4%) [18].

Blood cultures are positive in more than half of the patients with bacterial meningitis [13, 19]. In a prospective cohort analysis of 252 patients with neurolisteriosis, the pathogen was detected in 35 patients exclusively by blood culture [20]. Blood cultures - at least two sets of aerobic/anaerobic blood culture flasks - should therefore be obtained in any case before the start of antibiotic therapy. In patients with suspected meningococcal meningitis, if skin changes are present, microscopic and cultural detection of the pathogen can also be attempted from skin efflorescences.

The detection of bacterial antigens in the CSF using commercially available latex agglutination tests (e.g. antigen detection of *Neisseria meningitidis*, *Streptococcus pneumoniae*, *Haemophilus influenzae* type b and *Streptococcus agalactiae*) can supplement or confirm the result of a suspicion expressed on the basis of microscopy [21, 22]. However, a negative test result does not rule out an infection with the respective pathogen. Antigen detection can be helpful in the following situations:

- to confirm/clarify uncertain microscopic CSF findings and missing/unavailable pathogen detection using multiplex PCR.
- in the case of CSF with clear pleocytosis and negative microscopic findings as well as missing/unavailable pathogen detection by means of multiplex PCR.

#### Recommendation 3 (strong consensus)

If acute bacterial meningitis is suspected, we recommend to determine (i) CSF cell count and cell differentiation, (ii) CSF protein, and (iii) CSF lactate or CSF/serum glucose ratio.

#### Recommendation 4 (strong consensus)

A useful parameter for differentiating between acute bacterial meningitis and viral meningitis is serum procalcitonin, which is almost always elevated in acute bacterial meningitis. Therefore, if acute bacterial meningitis is suspected, we suggest that serum procalcitonin is determined.

#### Recommendation 5 (strong consensus)

To diagnose the pathogen in acute bacterial meningitis, we recommend to carry out a Gram stain and an attempt to identify the pathogen in culture from the cerebrospinal fluid. We suggest that multiplex-PCR (meningitis panel) is also used. The multiplex-PCR does not replace the other standard microbiological diagnostics (Gram staining and attempt to identify the pathogen in culture and determination of antibiotic susceptibility).

#### Recommendation 6 (strong consensus)

We recommend that at least 2 blood culture sets (each consisting of one aerobic and one anaerobic culture bottle) are taken from all patients with suspected bacterial meningitis before the start of antibiotic therapy.

### Imaging studies

#### Cranial imaging to rule out signs of increased intracranial pressure prior to lumbar puncture in the initial phase

Computed tomographic signs of increased intracranial pressure include in particular:

- Presence of an intracranial mass (e.g., subdural empyema, brain abscess, intracerebral hemorrhage) with midline displacement or herniation of brain tissue.
- Hydrocephalus.
- (Generalized) cerebral edema with reduced/abrogated grey and white matter differentiation.
- Narrowed external CSF spaces (cortical sulci, basal cisterns), compression of the ventricles.

In addition to the assessment of signs of elevated intracranial pressure, this initial CT also serves to rule out other, non-infectious, acute processes and to detect findings indicative of an inflammatory focus e.g. in the paranasal sinuses or the mastoids.

#### Diagnostic imaging of bacterial meningitis and its complications

While native CCT is initially sufficient for assessing the presence of imaging signs of increased intracranial pressure, MRI is superior to CT for detecting direct signs of bacterial meningitis and its complications.

Typical direct MRI signs of bacterial meningitis are [23, 24].

- Leptomeningeal and/or dural contrast enhancement.
- Inflammatory exudate (pus) in the cortical sulci and/or basal cisterns.

In addition, the following intracranial complications can be detected:

- Ventriculitis (ventricular empyema) with pus in the ventricles and ependymal contrast enhancement.
- Hydrocephalus (occlusus or malresorptivus).
- Cerebritis.
- Brain abscess.
- Subdural empyema.
- Ischemic stroke (possibly hemorrhagic transformation), e.g. in concomitant cerebral vasculitis, vasospasms or septic-embolic encephalitis.
- Septic sinus or cerebral venous thrombosis and its secondary complications (venous edema and/or hemorrhage).
- Intracranial hemorrhage (e.g. in coagulopathy).
- Generalized cerebral edema.

##### Recommendation 7 (strong consensus)

In the initial phase of the disease, if there is a clinical suspicion of increased intracranial pressure, we recommend CCT as the preferred imaging technique.

##### Recommendation 8 (strong consensus)

We suggest to perform a cerebral MRI if there are clinical signs that cannot be explained by imaging findings using CT, or if there is clinical deterioration during antibiotic therapy, or if CT findings regarding a primary focus of infection are unclear.

### Complementary investigations

About 20% of all patients with acute bacterial meningitis suffer from an ENT focus such as sinusitis, otitis media, or mastoiditis [25]. If an ENT focus is present, rapid surgical treatment of the infection has become established in most centers. Although there are no studies on the optimal timing of surgery, rapid surgery within the first 24 to 48 h is recommended in most case series [26, 27]. Depending on the medical history and the clinical findings, other infectious foci should also be considered (e.g. by chest X-rays, abdominal sonography/CT, echocardiography).

#### Recommendation 9 (strong consensus)

We recommend to carry out an ENT examination as soon as possible after the patient has been admitted.

### Course and complications

Around half of adult patients with bacterial meningitis develop neurological and/or systemic complications of varying degrees of severity in the acute phase of the disease (Table 2).

**Table 2:** Intracranial complications of acute bacterial meningitis in adults

Important neurological complications	
Brain edema	10–30%
Cerebrovascular complications: • cerebral arterial complications: vasculitis, vasospasm, focal cortical hyperperfusion, affection of cerebral autoregulation, septic sinus thrombosis and thrombosis of cortical veins	15–40%
Hydrocephalus	10–15%
Vestibulocochlear complications (hearing loss, vestibulopathy)	10–30%
Cranial nerve palsy	ca. 10%
Cerebritis	< 5%
Epileptic seizures	15–30%
Rare complications of bacterial meningitis: brain abscess, subdural empyema	

Common extracranial complications in the acute phase of bacterial meningitis are:

- septic shock.
- disseminated intravascular coagulopathy.
- adult Respiratory Distress Syndrome (ARDS).
- arthritis (septic and reactive).
- electrolyte disorders such as hyponatremia.
- syndrome of inappropriate ADH secretion (SIADH).
- cerebral salt-wasting syndrome or central diabetes insipidus.
- rhabdomyolysis.
- pancreatitis.
- spinal complications (e.g. myelitis or spinal vasculitis).

#### Recommendation 10 (strong consensus)

Due to the frequent occurrence of intracranial and systemic complications, we suggest that patients with acute bacterial meningitis are treated on an intensive care unit in the initial phase of the disease. In the case of a pronounced impaired consciousness, we suggest that this is done on an intensive care unit with experience in the treatment of patients with severe CNS diseases.

#### Recommendation 11 (strong consensus)

We recommend to look for signs of intracranial complications on initial imaging. These include hydrocephalus, generalized cerebral edema, cerebral ischemia, and sinus/vein thrombosis.

#### Recommendation 12 (strong consensus)

Transcranial doppler sonography may be considered as a screening test to identify patients with an increased risk of cerebral ischemia as a result of vasculopathy.

#### Recommendation 13 (strong consensus)

In survivors of acute bacterial meningitis, long-term complications are common. We suggest that appropriate examinations to identify long-term sequelae (in particular hearing impairment and cognitive deficits) are performed.

### Therapy

#### General procedure

Rapid initiation of treatment is important and determines outcome: clinical trials have shown that delayed initiation of antibiotic therapy is associated with an unfavorable outcome in acute bacterial meningitis [28–30].

To confirm or rule out the diagnosis of acute bacterial meningitis, a lumbar puncture (LP) with simultaneous serum collection (e.g. to determine the CSF-serum glucose index) is required (26). In individual cases with an increase in intracranial pressure and/or intracranial space-occupying processes, LP can theoretically endanger the patient. However, performing cerebral imaging (CT) prior to LP usually involves a significant time factor. In turns, beginning antibiotic therapy before LP reduces the chance of successfully identifying the pathogen [31, 32].

Contraindications for LP in patients with bacterial meningitis are rare. There is no reason to perform CT before LP in all patients with suspected acute bacterial meningitis. However, performing a CT before LP appears indicated if there are clinical signs of possible intracranial complications that are considered a contraindication for LP. The clinical signs include (i) severely impaired consciousness, (ii) new focal neurological deficits, and (iii) recent epileptic seizures.

In patients with severely impaired consciousness, patients with focal neurological deficits (e.g. hemiparesis), and patients who have recently developed epileptic seizures, a cranial CT should be carried out prior to the CSF examination with the question of increased intracranial pressure. If the CT findings do not contradict, a lumbar puncture should be performed as soon as possible.

##### Recommendation 14 (strong consensus)

We recommend starting antibiotic therapy as early as possible (if possible within 1 h after the patient arrives at the hospital). Delaying antibiotic therapy for more than 3 h after hospital admission should be avoided.

##### Recommendation 15 (strong consensus)

**Fig. 1:**
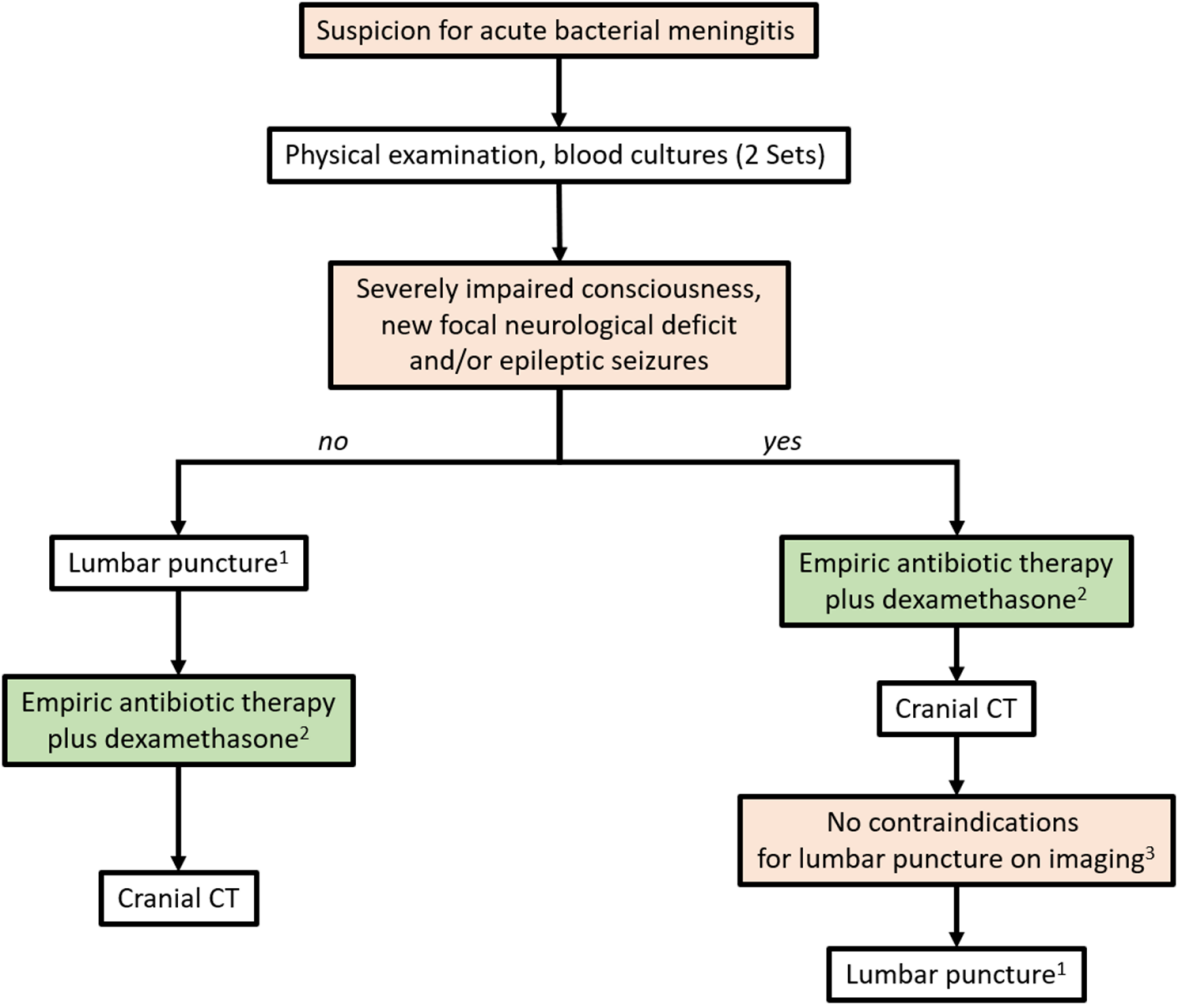
Workup in suspected bacterial meningitis (1) Other contraindications for lumbar puncture (such as manifest coagulation disorders, oral anticoagulation) need to be considered. (2) The beginning of antibiotic therapy plus dexamethasone is to be started within 1 h after arrival of the patient at the emergency department. A delay of more than 3 h should be strictly avoided. In case of any delay of the lumbar puncture, antibiotic therapy and adjunctive dexamethasone should be started before CSF is obtained. (3) Imaging signs that can hint at increased intracranial pressure are e.g. generalized brain oedema, hydrocephalus or space occupying brain abscess

In adult patients without impaired consciousness, without a focal neurological deficit and without current epileptic seizures, we recommend to obtain 2 blood culture sets (aerobic and anaerobic) immediately after the clinical examination, followed by LP (Fig. 1). We recommend that dexamethasone (10 mg) and antibiotics are administered immediately after LP.

In patients with severely impaired consciousness, new onset focal neurological deficits and in patients with recent epileptic seizures, we recommend imaging by CCT before LP. If there are no contraindications on CT, we recommend to continue with a lumbar puncture immediately.

##### Recommendation 16 (strong consensus)

Since clinical deterioration can occur in a few patients with bacterial meningitis despite a lack of contraindications on imaging after a lumbar puncture, we recommend close clinical monitoring of patients after lumbar puncture.

##### Recommendation 17 (strong consensus)

In any case of a delay in the lumbar puncture, e.g. due to the need to perform a CT before the puncture, we recommend to begin therapy with dexamethasone and antibiotics immediately after the collection of blood for blood cultures.

##### Recommendation 18 (strong consensus)

As long as Herpes simplex virus encephalitis is a possible differential diagnosis in the initial phase, we recommend to add aciclovir i.v.

##### Recommendation 19 (strong consensus)

If a parameningeal focus of inflammation (e.g. sinusitis, mastoiditis) is detected clinically (e.g. otitis media) or on CT as a possible cause of the bacterial meningitis, we recommend rapid surgery to clear the infectious focus.

#### Antibiotic therapy

The recommended empiric therapy depends on the clinical constellation (Table 3). The recommended duration of antibiotic therapy for bacterial meningitis depends on the response to therapy and the type of pathogen (Table 4, for dosage see Table 5). The recommended duration of treatment in uncomplicated cases is 7 to 10 days for *Haemophilus influenzae* meningitis, 10–14 days for pneumococcal meningitis and 7–10 days for meningococcal meningitis. Whether even shorter treatment periods are possible in meningococcal meningitis (as reported in a New Zealand study) is unclear and requires further clinical studies [33]. Listeria meningitis and meningitis caused by Gram-negative enterobacteria are often treated with antibiotics for 3 weeks or longer [34].

**Table 3:** Empiric antibiotic therapy in acute bacterial meningitis in adults

Clinical Condition	Recommended antiobitics
Community-acquired, no immunosuprression	cephalosporine group 3a^1^ plus ampicillin^2^
Post neurosurgery or post brain trauma	vancomycin plus meropenem *or* vancomycin plus ceftazidime^3^ (plus metronidazole in case of surgery that include mucous membranes)
Immunosuppression	vancomycin plus meropenem^4^
Shunt-infection	vancomycin plus meropenem or vancomycin plus ceftazidime

^1^E.g. ceftriaxone or cefotaxim. ^2^In regions with a high rate of cephalosporine-resistant pneumococci (e.g. France, Spain, Hungary, Australia, Southern Africa, Northern America initial treatment with a combination of ceftriaxone, vancomycin and ampicillin or ceftriaxone, rifampicin and ampicillin is recommended. ^3^or vancomycin plus cefepime. In cases of proven ventriculitis due to Staphylococcus aureus, intrathecal vancomycin can be considered [40]. ^4^Dependent on the clinical constellation, coverage of further pathogens (e.g. fungi) can be necessary

**Table 4:** Antibiotic therapy of acute bacterial meningitis in proven pathogens

Pathogen	Antibiotics^1^
*Neisseria meningitidis* ^*2*^	
- ceftriaxone/cefotaxim-susceptible (MIC ≤ 0,125 mg/l)	ceftriaxone^3^
- ceftriaxone/cefotaxim-resistant (MIC > 0,125 mg/l)	meropenem
*Streptococcus pneumoniae*	
- ceftriaxone/cefotaxim-susceptible (MIC ≤ 0,5 mg/l)	ceftriaxone^3^
	meropenem
- ceftriaxone/cefotaxim-resistant (MIC > 0,5 mg/l)	ceftriaxone^3^ + vancomycin,
	ceftriaxone^3^ + rifampicin^4^,
	Meropenem
*Haemophilus influenzae*	ceftriaxone^3^,
	ampicillin
Groupp-B-streptococci (*Streptococcus agalactiae*)	penicillin G,
	ceftriaxone,
	ampicillin,
	vancomycin
Gram-negative enterobacterales (e.g. Klebsiella spp., *E. coli*, Proteus spp.)	ceftriaxone^3^,
	meropenem,
	cefepime
*Pseudomonas aeruginosa*	ceftazidime,
	meropenem,
	cefepime
	(combination with fosfomycin^4^can be considered)
Staphylococci	
- methicillin-susceptible	cefazolin,
	flucloxacillin
	(combination with fosfomycin^4^ or
	rifampicin^4^ can be considered)
	vancomycin,
- Methicillin-resistant	linezolid^5^
	(combination with fosfomycin^4^ or
	rifampicin^4^ can be considered)
*Listeria monocytogenes*	ampicillin,
	trimethoprim-sulfamethoxazol,
	meropenem
	(combination with fosfomycin^4^ or
	rifampicin^4^ can be considered)

^1^The choice of the antibiotic depends on the susceptibility of the pathogen. ^2^During the last years, an increasing rate of resistances against penicillin was found for meningococci in Germany (2019: 5,9%, 2020: 3,9% und 2021: 8,5%, Nationales Referenzzentrum für Meningokokken und Haemophilus influenzae). In consequence, invasive meningococcal disease should not be treated with penicillin before the results of resistancy testing are available. However, as group A cephalosporins are usually effective against meningococci and as the results of resistancy testing are time consuming (usually taking several days), a possible benefit of changing the initially administered group A cephalosporine to penicillin seems questionable, even if the isolated pathogen is tested susceptible to penicillin. ^3^or cefotaxim. ^4^rifampicin, fosfomycin and aminoglykosides should not be given as monotherapy. ^5^linezolid is comparable ot vancomycin in terms of the covered pathogens and it penetrates well into the CSF; there are several reports on the use of linezolid in staphylococcal infections of the CNS [41, 42]. Linezolid should not be used as first-line medication. Its use should be considered when linezolid-susceptible bacteria are found causative for the CNS infection or (a) there are contraindications for vancomycin or vancomycin has to be stopped because of side effects, or (b) a clinical worsening is observed under therapy with vancomycin. In staphylococcal meningitis or ventriculitis, sufficient levels of linezolid are reached in the CSF [43], but as it is only moderately bactericidal, its use is of theroretical risk for the success of the therapy in meningitis

**Table 5:** Dosage of important antibiotics used in acute bacterial meningitis in adults

Antibiotic	Dosage per day
Penicillin G	6 × 5 Mio IE
Ampicillin	6 × 2 g
Cefotaxim	4 (bis 6) x 2 g
Ceftazidime	3 × 2 g
Ceftriaxone	1 × 4 oder 2 × 2 g
Meropenem	3 × 2 g
Fosfomycin	3 × 5 g^2^
Rifampicin	1 × 600 mg
Vancomycin	2 × 1 g^1^
Linezolid	2 × 600 mg
Gentamicin	1 × 240–360 mg^3^
Metronidazole	3 × 500 mg

^1^Determination of serum levels required. Cave: Dexamethasone might impair the penetration of vancomycin into the CSF [44]. ^2^Possibly, a fosfomycin-dosage of 3 × 8 g/d is needed to treat ventriculitis [45]. ^3^The daily gentamicin-dosis is 3–6 mg/kg KG. Determination of serum levels required

##### Recommendation 20 (strong consensus)

For adults with community-acquired acute bacterial meningitis we recommend empiric intravenous (i.v.) treatment with ceftriaxone 2 × 2 g/day plus ampicillin 6 × 2 g/day. In regions with a high proportion of cephalosporin-resistant pneumococci (or a corresponding travel anamnesis), we recommend additional treatment with i.v. vancomycin 2 × 1 g/day (guide value, determination of levels necessary for optimal dose determination) or i.v. rifampicin 1 × 600 mg/day.

### Adjunctive therapy

#### Dexamethasone

##### Recommendation 21 (consensus)

For Germany, Austria and Switzerland we recommend to treat adult patients with a clinical suspicion of community-acquired bacterial meningitis with dexamethasone in addition to antibiotics (dexamethasone 10 mg i.v., 4 times a day).

We recommend that dexamethasone is continued for 4 days in the case of identification of pneumococci or if pneumococcal meningitis remains probable (e.g. no pathogen detection but otogenic focus).

In meningococcal meningitis, the use of corticosteroids may be considered in the case of hearing impairment.

##### Recommendation 22 (strong consensus)

We suggest that dexamethasone is administered for the first time immediately before administration of the antibiotic (or at the same time). If antibiotic therapy has already started without corticosteroids, adjunctive dexamethasone therapy may be considered up to a few hours after the start of antibiotic therapy.

#### Other adjunctive therapeutic options

##### Recommendation 23 (strong consensus)

We do not recommend routine adjunctive therapy with glycerol or hypothermia in bacterial meningitis.

##### Recommendation 24 (strong consensus)

In the case of clinically relevant internal hydrocephalus, we recommend placement of an external ventricular drainage. In patients with reduced consciousness that is not explained otherwise, we suggest placement of an external ventricular drainage even without hydrocephalus on CT.

##### Recommendation 25 (strong consensus)

If there is increased intracranial pressure, we suggest to carry out measures to lower the intracranial pressure, e.g. by elevation of the upper body (30°), deep sedation, and an external ventricular drainage (EVD); in ventilated patients a short-term hyperventilation with a target pCO2 of 32–35 mm Hg may be considered. In addition, temporary osmotherapy with 20% mannitol i.v. may be considered.

##### Recommendation 26 (strong consensus)

In the case of vasculopathy, therapy with nimodipine and/or corticosteroids may be considered. If nimodipine is administered intravenously, we suggest intra-arterial blood pressure monitoring.

##### Recommendation 27 (strong consensus)

In the case of sinus vein thrombosis, we suggest therapy with intravenous heparin in the therapeutic range.

### Special considerations in meningococcal disease

#### Isolation of the patient, hygienic measures, chemoprophylaxis, vaccination

Meningococci are transmitted through close contact with an asymptomatic colonized individual or a diseased person via oropharyngeal secretions. The incubation period is usually 3–4 days (range 2–10 days). Patients with suspected meningococcal meningitis (e.g. petechial rash, gram-negative cocci in CSF Gram stain) must be isolated for up to 24 h after starting effective antibiotic therapy; after that, contagiousness is no longer to be expected (see also recommendations of the Robert Koch Institute, Internet address: www.rki.de). Close contact persons (e.g. close household members) must be reported to the appropriate health authorities and informed about the increased risk and possible symptoms of meningococcal disease (e.g. fever, chills, headaches). For close contact persons, chemoprophylaxis is recommended (Table 6). Chemoprophylaxis should be started as soon as possible; it makes sense for a maximum of 10 days after the last contact with the sick person. In the case of household contacts and close contacts of a household-like character, according to the recommendation of the RKI’s vaccination commission – if the index case fell ill with a vaccine-preventable serogroup – an additional post-exposure meningococcal vaccination should be carried out with a vaccine that covers the corresponding serogroup. This applies to serogroups A, C, W, Y and B [35–37] in Germany, Austria and, since May 2022, also in Switzerland. If meningococcal meningitis is treated with penicillin G, additional treatment with rifampicin, ciprofloxacin or ceftriaxone to eradicate the pathogens in the nasopharynx should be administered before discharge.

**Table 6:** Chemoprophylaxis of invasive meningococcal disease (recommendation of RKI, see “RKI-Ratgeber Meningokokken” at www.rki.de, Information downloaded 13.9.2022)

Antibiotics and age groups	Dosage
Rifampicin ^1,2^:	
Adolescents and adults > 60 kg	600 mg q 12h for 2 days p.o.
Infants, children and adolescents < 60 kg	10 mg/kg q 12h for 2 days p.o.
Newborns	5 mg/kg q 12h for 2 days p.o.
Ciprofloxacin ^2,3^:	
adults	500 mg single shot p.o.
Ceftriaxone ^4^	
adults and children ≥ 12 years	250 mg single shot i.m.
children < 12 years	125 mg single shot i.m.
Azithromycin ^5^	
adults	500 mg single shot p.o.

^1^see recommendations Robert-Koch Instituts, www.Rki.de, ^2^not in pregnant women, ^3^Only adults, not in pregnant or lactating women, ^4^ceftriaxone is considered first choice in pregnant women (according to RKI), ^5^As there is little evidence up to date and as there is a risk of the development of resistance, azithromycin should be preserved as an alternative for pregnant women

#### Reporting to health authorities

In Germany, suspected illness, illness and death from meningococcal meningitis or sepsis are notifiable in accordance with the Infection Protection Act (IfSG, § 6 Notifiable diseases). The report by name needs to be made by the treating physician within 24 h to the health department responsible for the affected person’s residence or stay. The person obliged to report must inform the health department immediately if a suspicion has not been confirmed.

## Data Availability

Literature was search using PubMed and Embase. Furthermore, data was acquired through the governments´ central scientific institutions in the field of biomedicine (for Germany, Austria, and Switzerland).

## Declarations

### Methodological approach

These guidelines were developed under the auspices of the German Neurological Society (*Deutsche Gesellschaft für Neurologie*, DGN) under guidance of *Arbeitsgemeinschaft der Wissenschaftlichen Medizinischen Fachgesellschaften* (AWMF). The development of the guideline took place in a nominal group technique within several guideline conferences. Final decisions were developed and agreed using a modified Delphi technique. All recommendations were voted on in a final consensus conference. The grading of the recommendation is expressed as follows: “we (do not) recommend” = strong recommendation, “we (do not) suggest” = recommendation, “may be considered” = open recommendation. Strength of consensus is expressed as follows: strong consensus = agreement > 95% of the participants, consensus = agreement > 75–95% of the participants, majority agreement = agreement of > 50–75% of the participants and no consensus = agreement of < 50% of the participants. Date of last update: April 28th, 2023; valid until: April 27th, 2028. AWMF registry number is 030/089.

## References

[1] van de Beek D, de Gans J, Spanjaard L, Weisfelt M, Reitsma JB, Vermeulen M (2004). Clinical features and prognostic factors in adults with bacterial meningitis. New England Journal Of Medicine.

[2] Bijlsma MW, Brouwer MC, Kasanmoentalib ES, Kloek AT, Lucas MJ, Tanck MW, van der Ende A, van de Beek D (2016). Community-acquired bacterial meningitis in adults in the Netherlands, 2006-14: A prospective cohort study. The Lancet Infectious Diseases.

[3] Domingo P, Pomar V, de Coll BN (2013). The spectrum of acute bacterial meningitis in elderly patients. Bmc Infectious Diseases.

[4] Cabellos C, Viladrich PF, Ariza J, Maiques JM, Verdaguer R, Gudiol F (2008). Community-acquired bacterial meningitis in cirrhotic patients. Clinical Microbiology & Infection.

[5] Koelman, D. L. H., van Kassel, M. N., Bijlsma, M. W., Brouwer, M. C., van de Beek, D., & van der Ende, A. (2020). : Changing epidemiology of bacterial meningitis since introduction of Conjugate Vaccines: Three decades of National Meningitis Surveillance in the Netherlands. *Clinical Infectious Diseases*. 10.1093/cid/ciaa1774 PMC8423501 33247582

[6] van de Beek D, Brouwer M, Hasbun R, Koedel U, Whitney CG, Wijdicks E (2016). Community-acquired bacterial meningitis. Nat Rev Dis Primers.

[7] Koelman DLH, Brouwer MC, van de Beek D (2020). Resurgence of pneumococcal meningitis in Europe and Northern America. Clinical Microbiology & Infection.

[8] Hoen B, Varon E, de Debroucker T, Fantin B, Grimprel E, Wolff M, Duval X (2019). Management of acute community-acquired bacterial meningitis (excluding newborns). Long version with arguments. Med Mal Infect.

[9] van Ettekoven CN, van de Beek D, Brouwer MC (2017). Update on community-acquired bacterial meningitis: Guidance and challenges. Clinical Microbiology & Infection.

[10] de Almeida SM, Furlan SMP, Cretella AMM, Lapinski B, Nogueira K, Cogo LL, Vidal LRR, Nogueira MB (2020). Comparison of cerebrospinal fluid biomarkers for Differential diagnosis of Acute Bacterial and viral meningitis with atypical cerebrospinal fluid characteristics. Medical Principles And Practice : International Journal Of The Kuwait University, Health Science Centre.

[11] Sakushima K, Hayashino Y, Kawaguchi T, Jackson JL, Fukuhara S (2011). Diagnostic accuracy of cerebrospinal fluid lactate for differentiating bacterial meningitis from aseptic meningitis: A meta-analysis. Journal Of Infection.

[12] Huy NT, Thao NT, Diep DT, Kikuchi M, Zamora J, Hirayama K (2010). Cerebrospinal fluid lactate concentration to distinguish bacterial from aseptic meningitis: A systemic review and meta-analysis. Critical Care.

[13] Koelman DLH, Brouwer MC, Ter Horst L, Bijlsma MW, van der Ende A, van de Beek D (2022). Pneumococcal meningitis in adults: A prospective Nationwide Cohort Study over a 20-year period. Clinical Infectious Diseases.

[14] Dubos F, Korczowski B, Aygun DA, Martinot A, Prat C, Galetto-Lacour A, Casado-Flores J, Taskin E, Leclerc F, Rodrigo C (2008). Serum procalcitonin level and other biological markers to distinguish between bacterial and aseptic meningitis in children: A european multicenter case cohort study. Archives Of Pediatrics And Adolescent Medicine.

[15] Krysan D (2009). Serum procalcitonin levels aid in distinguishing bacterial from aseptic meningitis in children. Journal Of Pediatrics.

[16] Prasad, R., Kapoor, R., Mishra, O. P., Srivastava, R., & Kant, S. U. (2013). : Serum procalcitonin in septic meningitis. *Indian Journal Of Pediatrics*. 10.1007/s12098-012-0933-3 23334585

[17] Viallon A, Desseigne N, Marjollet O, Birynczyk A, Belin M, Guyomarch S, Borg J, Pozetto B, Bertrand JC, Zeni F (2011). Meningitis in adult patients with a negative direct cerebrospinal fluid examination: Value of cytochemical markers for differential diagnosis. Critical Care.

[18] Trujillo-Gómez J, Tsokani S, Arango-Ferreira C, Atehortúa-Muñoz S, Jimenez-Villegas MJ, Serrano-Tabares C, Veroniki AA, Florez ID (2022). Biofire FilmArray Meningitis/Encephalitis panel for the aetiological diagnosis of central nervous system infections: A systematic review and diagnostic test accuracy meta-analysis. EClinicalMedicine.

[19] van de Beek D, Cabellos C, Dzupova O, Esposito S, Klein M, Kloek AT, Leib SL, Mourvillier B, Ostergaard C, Pagliano P (2016). ESCMID guideline: Diagnosis and treatment of acute bacterial meningitis. Clinical Microbiology & Infection.

[20] Charlier C, Poiree S, Delavaud C, Khoury G, Richaud C, Leclercq A, Helenon O, Lecuit M, Group MS (2018). Imaging of human neurolisteriosis: A prospective study of 71 cases. Clinical Infectious Diseases.

[21] Kniehl, E., Dörries, R., & Geiß, H. K. (2001). : Qualitätsstandards in der mikrobiologisch-infektiologischen Diagnostik. *Hrsg MiQ, Infektionen des Zentralnervensystems, München: Urban&Fischer*

[22] Saha SK, Darmstadt GL, Baqui AH, Hossain B, Islam M, Foster D, Al-Emran H, Naheed A, Arifeen SE, Luby SP (2008). Identification of serotype in culture negative pneumococcal meningitis using sequential multiplex PCR: Implication for surveillance and vaccine design. Plos One.

[23] Lummel N, Koch M, Klein M, Pfister HW, Bruckmann H, Linn J (2016). Spectrum and prevalence of pathological intracranial magnetic resonance imaging findings in Acute bacterial meningitis. Clinical Neuroradiology.

[24] Lummel, N., Koch, M., Klein, M., Pfister, H. W., Brückmann, H., & Linn, J. (2014). : Spectrum and prevalence of pahological intracranial magnetic resonance imaging findings in acute bacterial meningitis. *Clinicial Neuroradiology*. 10.1007/s00062-014-0339-x 25245328

[25] Ostergaard C, Hoiby N, Konradsen HB, Samuelsson S (2006). Prehospital diagnostic and therapeutic management of otogenic Streptococcus pneumoniae meningitis. Scandinavian Journal Of Infectious Diseases.

[26] Buchholz G, Koedel U, Pfister HW, Kastenbauer S, Klein M (2016). Dramatic reduction of mortality in pneumococcal meningitis. Critical Care.

[27] Kaplan DM, Gluck O, Kraus M, Slovik Y, Juwad H (2017). Acute bacterial meningitis caused by acute otitis media in adults: A series of 12 patients. Ear, Nose, And Throat Journal.

[28] Grindborg O, Naucler P, Sjolin J, Glimaker M (2015). Adult bacterial meningitis-a quality registry study: Earlier treatment and favourable outcome if initial management by infectious diseases physicians. Clinical Microbiology & Infection.

[29] Proulx N, Frechette D, Toye B, Chan J, Kravcik S (2005). Delays in the administration of antibiotics are associated with mortality from adult acute bacterial meningitis. Qjm.

[30] Koster-Rasmussen R, Korshin A, Meyer CN (2008). Antibiotic treatment delay and outcome in acute bacterial meningitis. Journal Of Infection.

[31] Michael B, Menezes BF, Cunniffe J, Miller A, Kneen R, Francis G, Beeching NJ, Solomon T (2010). Effect of delayed lumbar punctures on the diagnosis of acute bacterial meningitis in adults. Emergency Medicine Journal: Emj.

[32] Wylie, P. A., Stevens, D., Drake, W. 3rd, Stuart, J., & Cartwright, K. (1997). Epidemiology and clinical management of meningococcal disease in west Gloucestershire: Retrospective, population based study. *Bmj*, *315*(7111), 774–779. 10.1136/bmj.315.7111.774 PMC2127533 9345169

[33] Broom, M., Best, E., Heffernan, H., Svensson, S., Hansen Hygstedt, M., Webb, R., Gow, N., Holland, D., Thomas, M., & Briggs, S. : Outcomes of adults with invasive meningococcal disease with reduced penicillin susceptibility in Auckland 2004–2017. *Infection* 2022. 10.1007/s15010-022-01897-6 35982367

[34] Roos, K. L., Tunkel, A., van de Beek, D., & Scheld, M. : Acute bacterial meningitis. *Infections of the Central Nervous System* 2014:365–419.

[35] Ergänzung der Meningokokken-Impfempfehlung (2022). : Meningokokken-B-Impfung für Personen mit erhöhtem Erkrankungsrisiko. *BAG-Bulletin* 21.

[36] Bundesministerium (2022). : Impfplan Österreich 2022.

[37] RKI: *Epidemiologisches Bulletin* (2018). 3.

[38] Jaijakul S, Salazar L, Wootton SH, Aguilera E, Hasbun R (2017). The clinical significance of neutrophilic pleocytosis in cerebrospinal fluid in patients with viral central nervous system infections. International Journal Of Infectious Diseases : Ijid : Official Publication Of The International Society For Infectious Diseases.

[39] Wen L, Li M, Xu T, Yu X, Wang L, Li K (2019). Clinical features, outcomes and prognostic factors of tuberculous meningitis in adults worldwide: Systematic review and meta-analysis. Journal Of Neurology.

[40] Pfausler B, Spiss H, Beer R, Kampl A, Engelhardt K, Schober M, Schmutzhard E (2003). Treatment of staphylococcal ventriculitis associated with external cerebrospinal fluid drains: A prospective randomized trial of intravenous compared with intraventricular vancomycin therapy. Case Report Journal Of Neurosurgery.

[41] Ntziora F, Falagas ME (2007). Linezolid for the treatment of patients with central nervous system infection. Annals Of Pharmacotherapy.

[42] Rupprecht TA, Pfister HW (2005). Clinical experience with linezolid for the treatment of central nervous system infections. European Journal Of Neurology.

[43] Beer R, Engelhardt KW, Pfausler B, Broessner G, Helbok R, Lackner P, Brenneis C, Kaehler ST, Georgopoulos A, Schmutzhard E (2007). Pharmacokinetics of intravenous linezolid in cerebrospinal fluid and plasma in neurointensive care patients with staphylococcal ventriculitis associated with external ventricular drains. Antimicrobial Agents And Chemotherapy.

[44] Paris MM, Hickey SM, Uscher MI, Shelton S, Olsen KD, McCracken GH (1994). Effect of dexamethasone on therapy of experimental penicillin- and cephalosporin-resistant pneumococcal meningitis. Antimicrobial Agents And Chemotherapy.

[45] Pfausler B, Spiss H, Dittrich P, Zeitlinger M, Schmutzhard E, Joukhadar C (2004). Concentrations of fosfomycin in the cerebrospinal fluid of neurointensive care patients with ventriculostomy-associated ventriculitis. Journal Of Antimicrobial Chemotherapy.

